# The influence of adipocyte-derived stem cells (ASCs) on the ischemic epigastric flap survival in diabetic rats

**DOI:** 10.1590/ACB360907

**Published:** 2021-11-08

**Authors:** Cristina Pires Camargo, Marcia Saldanha Kubrusly, Julio Morais-Besteiro, Martim Conrad Harmsen, Rolf Gemperli

**Affiliations:** 1 PhD. Discipline of Plastic Surgery - Microsurgical and Plastic Surgery Laboratory (LIM-04) - School of Medicine - Universidade de São Paulo (USP) – Sao Paulo (SP), Brazil.; 2 PhD. Liver Surgery Transplant Laboratory (LIM-37) - School of Medicine - Universidade de São Paulo (USP) – Sao Paulo (SP), Brazil.; 3 PhD. Discipline of Plastic Surgery - Microsurgical and Plastic Surgery Laboratory (LIM-04) - School of Medicine - Universidade de São Paulo (USP) – Sao Paulo (SP), Brazil.; 4 PhD. Department of Pathology and Medical Biology - University Medical Center Groningen - University of Groningen – Groningen, Netherlands.; 5 Full Professor and Head. Plastic Surgery Division - Discipline of Plastic Surgery - Microsurgical and Plastic Surgery Laboratory (LIM-04) - School of Medicine - Universidade de São Paulo (USP) – Sao Paulo (SP), Brazil.

**Keywords:** Diabetes, Ischemia, Surgical Flaps, Mesenchymal Stem Cells, Rats

## Abstract

**Purpose:**

To assess the effects of adipocyte-derived stem cell (ASC)-injection on the survival of surgical flaps under ischemia in diabetic rats.

**Methods:**

Diabetes was induced in 30 male Wistar rats using streptozotocin (55 mg/kg). After eight weeks, epigastric flap (EF) surgery was performed. The animals were divided into control (CG), medium-solution (MG), and ASC groups. The outcomes were: the survival area (SA), the survival/total area rate (S/TR), and expression levels (EL) of genes: C5ar1, Icam1, Nos2, Vegf-a.

**Results:**

In the ASC group, compared to CG, we observed improved flap SA (CG-420 mm^2^ *vs*. ASC-720 mm^2^; p=0.003) was observed. The S/TR analysis was larger in the ASC group (78%) than the CG (45%). This study showed an increase in the Vegf-a EL in the ASC group (2.3) *vs*. CG (0.93, p=0.0008). The Nos2 EL increased four-fold in the ASC group compared to CG, and C5ar1 EL decreased almost two-fold in the ASC group *vs*. the CG (p=0.02). There was no difference among the groups regarding Icam1 EL. Compared to the MG, the ASC group had a bigger flap SA (720 mm^2^ *vs*. 301 mm^2^, respectively), a bigger S/TR (78% *vs*. 32%, p=0.06, respectively) and increased EL of Vegf-a (2.3 *vs*. 1.3, respectively). No difference between ASC-group and MG was seen regarding Nos2 (p=0.08) and C5ar1 (p=0.05).

**Conclusions:**

This study suggests that ASCs increase the survival of EF under IR in diabetic rats.

## Introduction

Several strategies have been developed to increase flap survival in patients with diabetes, *e.g.*, VEGF-a to stimulate neoangiogenesis^1^, and vasodilators^2^ and hyperbaric oxygen therapy to increase the oxygen supply^3^. However, these therapies have shown uncertain outcomes, because the necrosis may have multifactorial origin^2^^,^ 3.

In this sense, the use of stem cells in regenerative therapies can be an alternative^4^^,^ 5.

Mesenchymal cells can modulate inflammation (paracrine effect) and promote cell proliferation in injured tissues^6^.

Among mesenchymal stem cells, adipocyte-derived stem cells (ASCs) appear most promising, due to their low invasive harvesting and the possibility of collecting these in large quantities of liposuction^6^^-^ 9.

However, there is a lack of data related to the effects of ASCs on IR-induced tissue damage in diabetes.

Diabetes induces a pro-inflammatory state in the body. This activates the inflammation-related inducible nitric oxide synthase (Nos2) and causes uncoupling of the physiological NO balance. Inflammation renders vascular endothelial cells more adhesive to leukocytes through upregulation of intercellular adhesion molecule 1(ICAM-1). In diabetics, this endothelial dysfunction therefore also inhibits necessary angiogenic processes, that would augment tissue perfusion and wound healing. The administration of ASCs seems promising, because they act as a double-edged sword (immunomodulation and angiogenesis)^10^^-^ 13.

For this reason, this study aimed to evaluate the effects of ASCs on the survival of ischemic axial flaps in diabetic rats.

## Methods

This study was approved by the Ethical Committee of the School of Medicine, Universidade de São Paulo (050/16). All animal management was in accordance with the International Council for Laboratory Animal Science.

We analyzed 30 isogenic male Wistar rats weighting 250–300 g. The animals were kept in a vivarium on a 12-h day/night cycle and fed standard feed and water *ad libitum*.

### Diabetes induction

Streptozotocin (streptozotocin mixed anomers 031M1287V; Sigma-Aldrich, St. Louis, MO, USA) was injected (single dose) via the penile vein at a dosage of 55 mg/kg diluted in PBS (pH = 7) under inhalation anesthesia (20% isoflurane; 150-200 mL/min). Serum glucose levels were measured 24 h after the injections to confirm induction of diabetes (glycemia > 200 mg/dL). Afterwards, all animals were maintained for 8 weeks without any treatment (insulin injection), and had the glycemic level (mean 521 mg/dL) measured before receiving surgery.

### Harvesting and expanding ASC

Three male Wistar rats, weighting 220-250 g, 8 weeks old, were used in this procedure. The rats were anesthetized by intraperitoneal injection of ketamine (100 mg/kg) and xylazine (5 mg/kg). Both inguinal regions were trichotomized, and topical chlorhexidine was applied for antisepsis. An oblique 1.5 cm incision along the inguinal region was made. The panniculus carnosus was dissected, and adipose tissues near the femoral and inferior vessels were dissected. The fat was placed in PBS solution and washed three times to remove blood and debris. It was cut using iris scissors and tweezers, and the pieces (<1 mm) were enzymatically dissociated for 30 min at 37°C in 0.1% trypsin/EDTA (Sigma-Aldrich). The trypsin was then inactivated by 10 mL of Dulbecco’s modified Eagle’s Medium (DMEM) supplemented with 10% fetal bovine serum (FBS) and 1% penicillin and erythromycin.

The digested material was centrifuged at 363 g for 5 min at 20°C to obtain cell pellets. The supernatant was carefully removed with a Pasteur pipette. The pellets were washed with 10 mL of DMEM.

After centrifugation, the supernatant was discarded and the pellet resuspended in 2 mL of DMEM, and 10 μL of the suspension was used to determine cell survival by counting in a Neubauer chamber after staining with 0.4% trypan blue dye.

The cell suspensions were adjusted to 5 mL, placed in 25 cm^2^ tissue culture flasks, and stored in a culture incubator under a humid atmosphere containing 5% CO_2_ at 37°C. Cells were passaged at >80% confluence. An enzymatic dissociation method with 0.25% trypsin/EDTA in PBS for 2 min at 37°C was used to detach cells from the culture bottles (1:3 ratio). The cells were expanded (1:3) to passage 4 and frozen in liquid nitrogen.

For cell freezing, the culture medium was removed, and enzymatic digestion was performed with 0.25% trypsin/EDTA for 2 min at 37°C. Digestion was terminated with DMEM supplemented with 10% FBS, and the cell suspensions were centrifuged at 1,800 rpm for 5 min. The resulting cell pellets were washed twice with DMEM. After the supernatant was discarded, the pellets were resuspended in 2 mL of 10% FCS for cell counting in a Neubauer chamber and subsequent calculation for a final concentration of 1×10^6^ cells/mL in PBS in cryogenic tube. After this procedure, we confirmed the cell viability and mesenchymal nature by flow cytometry^14^.

### Surgical and ischemia/reperfusion procedures

**Figure 1:**
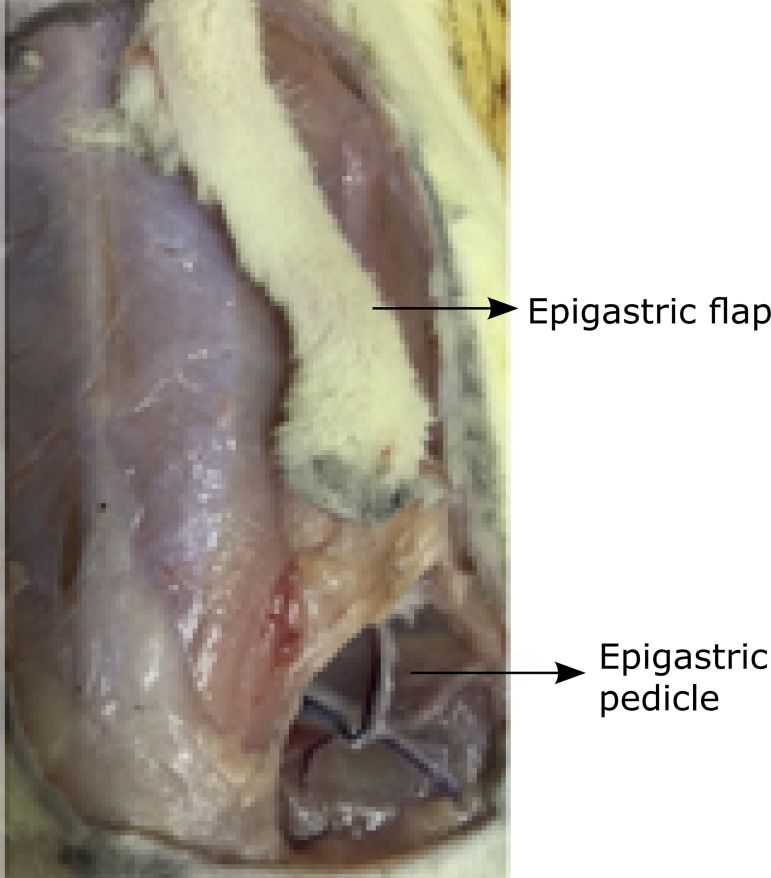
Epigastric pedicle and surgical flap.

All animals were anesthetized with intraperitoneal injections of 100 mg/kg ketamine hydrochloride (Ketalar®; Parke Davis, Detroit, MI, USA) and 15 mg/Kg xylazine (Rompun® 2%; Bayer, Leverkusen, Germany). The ventral face of the abdomen was trichotomized. A 6 × 3-cm flap based on the inferior epigastric pedicle (IEP) was designed (Fig. 1). The flap was harvested, and a vascular clamp was applied in the IEP for 3 h.

The vascular clamp was released, and 1 mL of medium-solution (MG) or ASC (1×10^6^ cells) was injected subcutaneously according to group allocation. The flap in the original location was sutured using mononylon 4-0 (Ethicon®, J &J, United States).

After the surgical procedure, the animals were then allocated into three groups:

- Control group (CG) (n=10);
- Medium-solution group (MG – DMEM supplemented with 10% FBS) (*i.e.*, without ASC) (n=10);
- ASC group (ASC) (n=10).

### Analysis of the survival area and survival area/total area rate

On the seventh postoperative day, all animals were euthanized by anesthetic overdose.

All flaps were photographed along with a centimeter ruler. The images were transferred to ImageJ®^15^, and the total and survival areas (mm^2^) were measured. These data were entered into a Microsoft Excel® (Microsoft Corporation, Redmond, WA, USA) spreadsheet, and the ratios of survival to total area were calculated.

### Microscopic analysis

We collected a stripe of tissue (0.5-cm wide in the longitudinal axis of the flap). This sample was immersed in 4% neutral, phosphate buffered, paraformaldehyde, for 48 h at room temperature.

Tissue was washed, dehydrated in graded concentrations of alcohol, and embedded in paraffin. Four-micrometer-thick sections were mounted on glass slides and stained with hematoxylin-eosin (HE). The sections were analyzed under the light microscope Nikon Optiphot-2 (Nikon, Tokyo, Japan), coupled to a Nikon DXM 1200F® (Nikon, Tokyo, Japan) video digital camera. Measurements were performed using the ImageJ® (Media Cybernetics, Silver Spring, MD, USA). A blinded investigator counted arterioles, inflammatory cells, and dermic appendages in ten fields per specimen (x20).

### Analysis of Vegf-a (angiogenesis), Nos2 (ischemia), Icam-1 (cell adhesion), C5ar1 (complement system)

#### RNA isolation

Skin samples maceration was performed with a tissue Lyser LT® (Qiagen, Hilden, Germany). The products were microcentrifuged (10,000×g) with 1 mL of Trizol® (Invitrogen, Carlsbad, CA, United States) and stainless-steel beads. Fragmentation was performed for 6 min at 50 Hz.

After removal of the beads, 0.2 mL of chloroform (Merck, Whitehouse Station, NJ, United States) was added. The samples were centrifuged for 15 min at 10,000×g and 4°C. The aqueous phase was then transferred to a new microcentrifuge tube, and 0.5 mL of ice-cold isopropanol (Merck) was added to precipitate the RNA. Samples were incubated for 10 min and then centrifuged at 10,000×g for 10 min at 4°C. The supernatant was discarded, and the precipitated RNA washed with 1 mL of 75% ethanol. The RNA was then centrifuged for 5 min at 10,000×g and 4°C. The RNA pellet was resuspended in 50–100 μL of DNase/RNase-free sterile ultrapure water (Invitrogen).

The concentration of extracted RNA was determined using a NanoDrop™ ND-1000 spectrophotometer (Thermo Fisher Scientific, Waltham, MA, United States). Purity was evaluated by the absorbance ratio at 260/280 nm, using only the RNAs whose ratios were ≥ 1.8. To analyze RNA integrity, agarose gel electrophoresis was performed to verify the 28S and 18S bands. The extracted RNAs were stored at -80°C.

#### cDNA synthesis

A high-capacity RNA-to-cDNA kit (Applied Biosystems, Foster City, CA, USA) and GeneAmp 2400 thermocycler (Applied Biosystems) were used for the synthesis of cDNA from total RNA. For reaction and inactivation of this reaction, the tubes were incubated at 37°C for 60 min and at 95°C for 5 min, respectively. The cDNA samples were stored at -20°C until use.

### Reverse transcription semi-quantitative polymerase chain reaction

Analysis of mRNA expression of the genes of interest was performed by reverse transcription semi-quantitative polymerase chain reaction (qRT-PCR) in a StepOnePlus™ thermocycler (Applied Biosystems) with the TaqMan® gene expression assay system (Applied Biosystems). The probes and primers for the genes (rats) C5ar1 (Rn02134203_s1), Icam1 (Rn00564227_m1), Nos2 (Rn 00561646_m1), and Vegf-a (Rn01511602_m1) and for the endogenous control Actb (Rn 00667869_m1) were purchased from Applied Biosystems. qRT-PCR was performed in duplicate for each sample using 10 μL TaqMan® Universal Master Mix II 2X, 1 μL TaqMan® Gene Expression Assay 20×, and 4-μL diluted cDNA (1:5 dilution) for a final volume of 20 μL in 96-well plates coated with optical sealant. The reaction conditions were 50°C for 2 min, 95°C for 10 min, 40 cycles at 95°C for 15 s, and 60°C for 1 min.

The expression level of each target gene was calculated by GenEx Standard 6.1 (MultiD Analyses AB, Göteborg, Sweden), which uses the 2^-ΔΔ^Ct method for relative quantification, in which:

Ct (threshold cycle) = the point at which amplification reaches the logarithmic phase;

ΔCt = the difference in expression between the target gene and endogenous control of a given sample;

ΔΔCt = the difference between the ΔCt of the sample and the ΔCt of the control.

### Statistical analysis

Nonparametric variables are shown as medians and interquartile ranges. Multiple groups were compared using Kruskal-Wallis test. When comparison test had a significant p-value, pair-wise comparison was performed by Dunn’s test. Because of the small sample size, a bootstrap test was performed to certify the significance of our outcomes between CG and ASC group. The Hedges’ g statistic coefficient was used to calculate the effect size between the control and ASC groups. Analysis was performed in Stata v14 (StataCorp, College Station, TX, USA) with p-value and power thresholds of 5 and 80%, respectively.

## Results

### Analysis of the survival area, and survival area/total area rate

**Figure 2:**
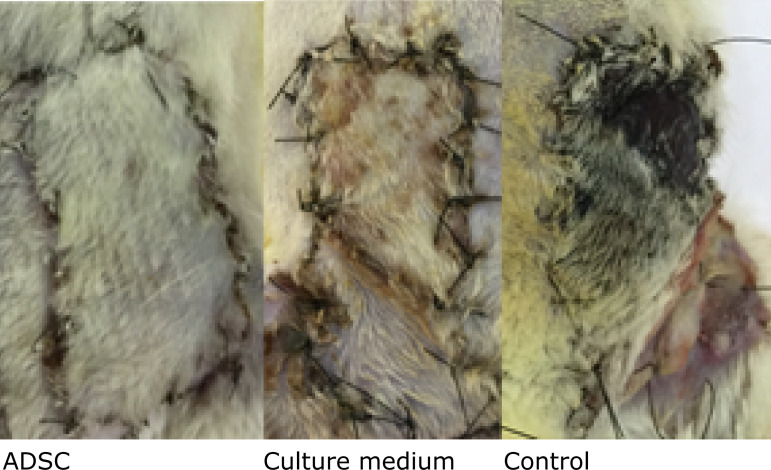
The epigastric flap area on the seventh postoperative day.

The survival area and ratio of survival to total area are shown in Table 1 and Fig. 2.

**Table 1:** The survival area and the ratio of survival to total area in all groups presented as medians and interquartile ranges.

Group	Survival area * (mm^2^) Median (IQR)	Rate SA/TA Median (IQR)
Control	420.00 (311.34–531.33)	0.45 (0.26–0.63)
Culture medium	301.76 (229.92–341.22)	0.32 (0.20–0.36)
ASC	720.32 (640.84–840.06)	0.78 (0.66–1)

* CG *vs*. MG, p=0.06; ASC group *vs*. CG, p=0.02; ASC group *vs.* MG, p<0.001; **ASC group *vs*. CG, p=0.003; MG *vs*. ASC group, p<0.001; SA/TA: survival area/total area; IQR: interquartile ranges; ASC: adipocyte-derived stem cell.

ASC: adipocyte-derived stem cell.

ASC: adipocyte-derived stem cell.

The comparison of survival area among the groups showed significant difference (p=0.0008). Pair-wise comparison showed a similar effect regarding the survival area between the control and MG (420 mm^2^ *vs*. 301.8 mm^2^; p=0.06, respectively).

The ASC group showed a higher survival area compared to CG (720 mm^2^ *vs*. 420 mm^2^, p=0.02).

The ASC treatment improved the survival area when compared to CG and MG groups (p <0.001). In summary, the treatment with ASC increase two-times the survival area when compared to CG and MG (Table 1).

We tested a bootstrap analysis of survival comparing the CG *vs*. the survival area of the ASC group (p<0.001, 95% confidence interval – 95%CI -3.02 to -0.99). The effect size with a coefficient of Hedges’ g statistic was -2.01.

The comparison of survival area/total area rate (S/TR) among the groups showed significant difference (p=0.008). A *post-hoc* test showed an increase in the S/TR between the CG and ASC group (45% *vs*. 78%, p=0.003, respectively) and between the MG group and ASC group (32% *vs*. 78%; p<0.001, respectively) (Table 1).

The comparison between CG and MG, regarding S/TR, showed no difference (p= 0.052) (Table 1).

### Microscopic analysis

The histology (H&E) of the CG and MG distal flap area showed full-thickness skin necrosis, loss of dermic structure, subcutaneous edema, and a large number of infiltrated inflammatory cells in all those flaps that suffered 3 h of ischemia.

**Figure 3:**
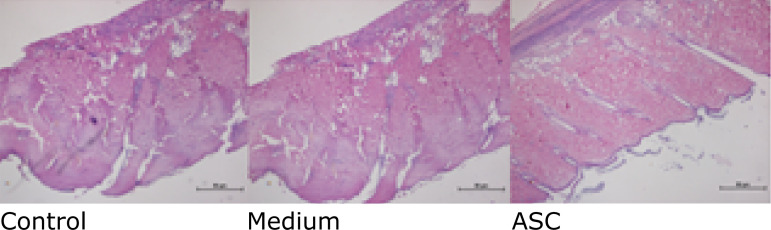
Histologic analysis: control group, medium group, and ASC group (magnification of x40).

The ASC group showed less inflammatory cell infiltration, less subcutaneous edema, and more neoangiogenesis when compared to the other groups (Fig. 3).

ASC: adipocyte-derived stem cell.

### Analysis of Vegf-a (angiogenesis), Nos2 (ischemia), Icam-1 (cell adhesion), and C5ar1 (complement system)

There were significant differences in Vegf-a expression between all groups (p=0.007).

A *post-hoc* analysis showed a 2.5-fold increase in the Vegf-a expression in the ASC group *vs.* the CG (2.3 *vs.* 0.93, p=0.0008, respectively). A similar result was observed when comparing Vegf-a expression in the ASC group *vs.* MG (p=0.03). This study showed similar results in Vegf-a expression between CG and MG (p=0.08) (Table 2).

**Table 2:** Median and interquartile ranges of Vegf-a, Nos2, Icam-1, and C5ar1 expression in all groups normalized to that of ACTB.

Group	Vegf-a Median (IQR)	Nos2 Median (IQR)	Icam-1 Median (IQR)	C5ar1 Median (IQR)
Control	0.93 (0.8–1.1)	0.85 (0.4–2.3)	1.01 (0.8–1.3)	0.95 (0.7–1.2)
Culture medium	1.3 (1.1–1.6)	11.2 (3.5–53.0)	0.64 (0.5–0.9)	0.81 (0.5–1.2)
ASC	2.3 (1.9–2.8)	3.4 (2.5–35.6)	0.93 (0.8–1.6)	0.5 (0.4–0.7)

Vegf-a: ASC group *vs*. the CG, p=0.0008; ASC group *vs*. MG, p=0.03; Nos2: ASC group *vs*. CG, p=0.03; MG *vs*. CG, p=0.001; C5r1: ASC group *vs*. the CG, p=0.02; MG *vs*. the ASC group, p= 0.05; IQR: interquartile ranges; ASC: adipocyte-derived stem cell.

Regarding Nos2 expression, there was a difference among the groups (p=0.009). A *post-hoc* test showed a four-fold increase in Nos2 expression when comparing to the ASC group and the CG (p=0.03), as well as an increase in Nos2 expression between the MG and CG (p=0.001), but no difference between ASC group and MG (p=0.191).

There were no differences in Icam 1 expression among the groups (p=0.06). Furthermore, there were significant differences in C5ar1 expression between all groups (p=0.002). The pair-wise comparison showed 50% of decrease in C5ar1 expression in the ASC group *vs*. the CG (p=0.02). A similar result was observed when comparing the MG and the ASC group (p= 0.05). There was no difference between CG and MG (p=0.143) (Table 2).

## Discussion

In the ASC group, we observed an increased survival area (720 mm^2^) when compared to the CG (420 mm^2^). In the ASC group, an increase of 1.8 times of the survival area/totalarea rate in the axial surgical flap when compared to the CG was also noticed. Similarly, Gao *et al*.^12^ showed an increase in the survival area in ASC group when compared to no treatment and MG in a random flap.

The flap survival depends on vascular regeneration. The histologic analysis showed an increase in vascular density and decreased inflammatory cells, edema, and necrosis in the ASC group compared to the control and culture medium group. These findings were similar to several authors’ ones^7^^,^ 8. The literature data hypothesized the increase of survival ratio in the ASC group because of the paracrine effect of the stem cells^8^^,^ 16.

Moreover, the effect of ASC treatment might be analyzed in the different phases of the ischemia/reperfusion phenomenon.

This study showed an increase in the VEGF-a levels in the ASC group compared with the control and medium groups. These findings were similar to the literature review by Foroglou *et al*.^6^ It showed an association of ASCs increased the vascular density and the surgical flap viability and cytokines (Vegf-a). In addition, Moritz *et al*.^17^ injected Vegf in surgical flaps submitted to ischemia-reperfusion and compared it with placebo. This experiment showed the positive effect of the Vegf-a cytokine on the viability of ischemic surgical flap^10^. Our results showed a four-fold increase in Nos2 levels in the ASC group compared with the control group (3.4 *vs*. 0.85, respectively, p=0.001).

There was a discussion about the possible role of NO in tissue regeneration. Kane *et al*.^18^ analyzed ischemic surgical flaps in iNos knock-out mice, and discussed the role of iNos in the wound healing process. Initially, iNos was responsible for decease inflammation in the wound site, but the authors demonstrated iNos has a role in the angiogenesis phase. Therefore, a higher level of these biomarkers (Vegf-a and Nos2) could work in synergy and explain the increase in the survival area^17^.

Adhesion molecules (Icam-1 and Vcam-1) reflect the reperfusion phase of inflammatory reaction to ischemia in which leukocytes adhere to the endothelium, increasing vessel permeability, amplifying the inflammatory reaction by the migration of more inflammatory cells. We expected A lower Icam-1 expression in the ASC group. However, we found no differences in the Icam-1 levels among the groups. We did not find No other studies that evaluated the effect of ASC treatment in Icam-1 levels. Song *et al*.^19^ analyzed the effect of hyperbaric oxygen therapy on Icam-1 and Vcam-1 levels in the abdominal skin flap submitted to ischemia-reperfusion and showed reduction in the treatment group.

The last stage of IR is the complement cascade activation. The cascade initiates by C1q, and the final product is C5. In this study, it was hypothesized that lower levels of complement in the ASC group could reflect the anti-inflammatory effect of ASC^20^. In fact, the ASC treatment showed 50% of decreased in the C5ar1 levels when compared with the control group (0.5 *vs*. 0.95, respectively, p=0.02) and with the MG group (0.5 *vs*. 0.81, respectively, p= 0.05). We hypothesized it was thought the treatment of ASC in ischemia and reperfused flap could minimize the tissue and cellular damage by the inflammatory and immunological response.

This study has some limitations. The ideal animal model for flap studies is porcine, but we used a murine model due to the more manageable size of the rat and to the existing literature. Due to the lack of literature analyzing biomarkers in diabetes with IR, we selected several biomarkers that may not be useful tools for assessing flap survival were selected. Some studies have investigated other biomarkers such as superoxide dismutase, and catalase, but the results have been inconclusive^21^^-^ 23.

To solve the small sample size power limitation, the significance of these findings was confirmed testing bootstrap analysis. This statistical tool was used to confirm whether the calculated p-value remained significant (p <0.001, 95%CI -3.02 to -0.99). In an attempting to explain the mechanism of action of ASC therapy, we analyzed surrogate endpoints.

Several comorbidities such as diabetes, cardiovascular disease, and smoking can interfere in wound healing process. In a free flap transfer procedure, the surgeon can add more factors to interfere in the tissue regeneration. The idea to use an autologous strategy to improve tissue regeneration is attractive. In this study, we injected ASC immediately after the IR to minimize tissue necrosis.

While we adopted ASC as a regenerative strategy on the survival of axial flap in the present study, it is recognized that stromal vascular fraction (SVF) has been increasingly studied in this arena^21^^-^ 24. The SVF is apparently less costly and technically less complicated than ASC^23^^,^ 24. In addition, SVF seems to be more available worldwide compared with ASC. In this sense, a comparative study of ASC *vs*. SVF would be of interest^22^^-^ 24.

## Conclusion

This study suggests that ASCs treatment increases the survival of axial flaps submitted to IR in diabetic rats.
